# Effects of quercetin and daidzein on egg quality, lipid metabolism, and cecal short-chain fatty acids in layers

**DOI:** 10.3389/fvets.2023.1301542

**Published:** 2023-12-22

**Authors:** Jiayan Liu, Junhong Liu, Shuaishuai Zhou, Yuxin Fu, Qinglin Yang, Yao Li

**Affiliations:** College of Animal Science and Technology, Northeast Agricultural University, Harbin, China

**Keywords:** quercetin, daidzein, layer, egg quality, nutritive value

## Abstract

In this study, the effects of quercetin and daidzein on egg quality, lipid metabolism, and cecal short-chain fatty acids (SCFAs) were compared in layers. Hyline brown layers at 385 days of age with a similar laying rate (81.36% ± 0.62%) and body weight (2.10 kg ± 0.04 kg) were randomly divided into three treatments, six replicates per treatment, and 20 layers per replicate. Layers in control, quercetin, and daidzein treatment were fed by a basal diet supplemented with 0 mg/kg, 500 mg/kg quercetin, and 30 mg/kg of daidzein for 10 weeks. Results showed that eggshell strength and albumen height in week 4, egg yolk diameter in week 10, and eggshell thickness and egg yolk height in weeks 4 and 10 were significantly increased in the quercetin treatment (*P* ≤ 0.05); contents of phospholipid (PL) and lecithin (LEC) in egg yolk and high-density lipoprotein (HDL) content in serum were significantly increased; however, contents of malondialdehyde (MDA), total cholesterol (TC), and triglyceride (TG) in egg yolk, contents of TC, TG, low-density lipoprotein (LDL), and very-low-density lipoprotein (VLDL) in serum, and contents of TC and TG in the liver were significantly decreased in the quercetin treatment (*P* ≤ 0.05); contents of isobutyric acid and valeric acid were significantly increased in the cecum of the quercetin treatment (*P* ≤ 0.05), compared with control. Moreover, egg yolk height in week 10 and eggshell thickness in weeks 4 and 10 were significantly increased in the daidzein treatment (*P* ≤ 0.05); contents of MDA, TC, and TG in egg yolk, TC, TG, and VLDL in serum, and TC and TG in liver were significantly decreased in the daidzein treatment (*P* ≤ 0.05); and HDL content was significantly increased in serum of the daidzein treatment (*P* ≤ 0.05) compared with control. However, daidzein did not affect SCFA content in the cecum. In conclusion, egg quality was improved by quercetin and daidzein by increasing the antioxidant ability of egg yolk and by regulating lipid metabolism in layers. Quercetin worked better than daidzein in improving egg quality under this experimental condition.

## 1 Introduction

Eggs have a high nutritive value and are one of the important food sources for humans (1, 2). Human demand for eggs has been increasing as the world's population increases. Highly intensive farming meets the demand of consumers; however, long-term and high-intensive laying may affect the egg quality, decrease the nutritive value of eggs, increase the number of broken eggs, and cause economic loss. Therefore, it is crucial to improve egg quality (3). For the past decades, the application of different safe feed additives has worked very well for reducing cholesterol deposition in egg yolk, improving eggshell thickness and egg quality, including vitamins and minerals (4), probiotics (5), amino acids (6), and flavonoids (7). Recently, flavonoids caught much attention as functional additives to improve product quality and enhance the economic benefit of animal husbandry.

Quercetin, a flavonoid rich in apples, onions, sea buckthorn, and hawthorn, may exhibit anti-inflammatory, anti-viral, and anti-oxidative effects (8, 9). Moreover, quercetin may regulate lipid metabolism and reduce hepatic lipid deposition in mice fed with a high-fat diet (10). Our previous studies found that 0.04% quercetin significantly decreased the contents of TC and TG and increased the contents of PL and LEC in egg yolk of laying hens, and 0.02% of quercetin increased eggshell thickness, thus demonstrating the positive effect of quercetin on egg quality (11–13). Daidzein is an isoflavone found in alfalfa, red clover, white clover, soybean, and other legumes (14). For the past few years, daidzein has been used in stock farming, because it acts like estrogen, increasing animal fertility and improving the quality of animal products. Dietary supplementation with 200 mg/kg of daidzein increased reproductive performance, antioxidant ability, and serum hormones of sows (15, 16). Dietary supplementation with daidzein enhanced production performance and serum antioxidant levels in cattle; moreover, daidzein also improved the meat and milk quality in cattle (17). A diet supplemented with daidzein improved follicle development and increased egg weight, eggshell strength and thickness, and calcium content in the eggshell of layers (18, 19).

Egg quality may be improved by quercetin and daidzein in layers. However, our previous study found that the price of daidzein limited its uses, and quercetin increased production performance and economic returns better than daidzein in laying hens (20). Meanwhile, few studies compare the effects of quercetin and daidzein on the egg quality of layers. Therefore, this study further investigated the effects of a diet supplemented with separate quercetin and daidzein on egg quality, lipid metabolism, and cecal short-chain fatty acids in aged layers based on the above-mentioned research (20). This will provide a theoretical foundation for using quercetin to improve the egg quality of aged layers.

## 2 Materials and methods

All procedures used in this study were approved by the Animal Care and Use Committee of the Northeast Agricultural University (NEAUEC20200203). Housing, management, and care of the birds conformed to the guidelines of Agricultural Animal in Agricultural Research and Teaching of Heilongjiang Province (HEI Animal Management Certificate No. 11928).

### 2.1 Experimental birds and diets

After 1 week of adaptation, Hyline Brown layers at 385 days of age with a similar laying rate (81.36% ± 0.62%) and body weight (2.10 kg ± 0.04 kg) were randomly divided into three treatments, six replicates per treatment, 20 layers each replicate. A single-factor experimental design was used in this study; layers in control, quercetin, and daidzein treatment were fed by a basal diet supplemented with 0 mg/kg, 500 mg/kg quercetin, and 30 mg/kg daidzein for 10 weeks. The basal diet was prepared by referring to the GB/T 5916-2020 Chinese Layers Feeding Standards (Table 1). Quercetin (CAS: 6151-25-3, purity ≥97%) was purchased from Nanjing Dulai Biotechnology Co. Ltd. (Nanjing, China), and daidzein (CAS: 468-66-8, purity ≥98%) was bought from Meryer (Shanghai) Chemical Technology Co. Ltd. (Shanghai, China). All layers were raised in triple wiry cages (526 × 423 × 381 mm; two birds in each cage) with 16-h natural light and artificial light per day in the experimental site of Northeast Agricultural University (Harbin, China) and were maintained with optimal ventilation during the experimental period. Layers had access to water and feed *ad libitum* during the 10-week experimental period.

**Table 1:** Composition and nutrient levels of basal diet (air-dry basis).

**Ingredients**	**Composition (%)**	**Nutrient levels**	**Nutrient content**
Corn meal	62.72	ME (MJ/kg)	11.15
Soybean meal	27.50	CP (%)	17.04
Calcium hydrogen phosphate	1.50	Lys (%)	0.89
Limestone	7.50	Met + Cys (%)	0.56
Sodium chloride	0.32	Ca (%)	3.20
Met	0.15	TP (%)	0.58
Vitamins premix	0.02	AP (%)	0.37
Choline	0.09		
Minerals premix	0.20		
Total	100.00		

Per-kilogram of diet provides vitamin A, 10,000 IU; vitamin D, 3,000 IU; vitamin E, 12 IU; vitamin K, 3 mg; vitamin B_1_, 2 mg; vitamin B_2_, 6 mg; vitamin B_6_, 3 mg; vitamin B_12_, 0.012 mg; biotin 0.04 mg; folic acid, 0.6 mg; niacin, 20 mg; pantothenic acid, 10 mg; chromium picolinate, 0.6 mg; Cu (CuSO_4_•5H_2_O), 10 mg; I (KI), 0.35 mg; Fe (FeSO_4_•7H_2_O), 65 mg; Mn (MnSO_4_•H_2_O), 120 mg; Se (NaSeO_3_), 0.25 mg; Zn (ZnO), 65 mg. Values of nutrient levels were calculated.

ME, metabolic energy; CP, crude protein; TP, total phosphorus; AP, available phosphate; Lys, lysine; Met, methionine; Cys, cysteine.

### 2.2 Egg quality

At the end of weeks 4 and 10 of the experiment, 30 eggs/treatment were randomly collected to determine egg quality. The egg weight (g), albumen height (mm), egg yolk color, and Haugh unit were determined using an egg multi-tester (EMT-5200, Robotmation, Japan). The eggshell strength (*N*) was determined using eggshell strength meter (FHK-700 IIDP, Japan). The egg yolk height (mm) and diameter (mm) were determined using vernier calipers. The eggshell thickness was measured by the average of the sharp end, middle section, and blunt end of the eggshell using micrometer calipers.

### 2.3 Sample preparation

At the end of the experiment, 30 eggs/treatment were randomly broken, and egg yolks were separated and stored at −80°C. The blood samples were collected from the jugular vein of six layers selected randomly and euthanized by cervical dislocation in each treatment (one layer per replicate) (21), and then centrifuged at 3,000 r/min for 15 min to obtain the serum and stored at −80°C. After blood collection, the layers were dissected, and the liver and fresh cecal digesta were taken, quickly frozen in liquid nitrogen and stored at −80°C for the following analysis.

### 2.4 Serum, liver, and egg yolk indices

The contents of malondialdehyde (MDA), total cholesterol (TC), triglyceride (TG), phospholipid (PL), lecithin (LEC) in egg yolk; the contents of TC, TG, low-density lipoprotein (LDL), high-density lipoprotein (HDL), and very-low-density lipoprotein (VLDL) in serum; and the contents of TC and TG in the liver were determined using enzyme-linked immunosorbent assay (ELISA) following the kit instructions purchased from Jiangsu Baolai Biotechnology Co. Ltd. (Jiangsu, China).

### 2.5 Cecal short-chain fatty acids

Appropriate samples were put in 2-ml centrifuge tubes; 50 μl of 15% phosphoric acid, 100 μl of 125 μg/ml isocaproic acid, and 40 μl of ether were added in sequence for 1 min and then centrifuged at 12,000 r/min for 10 min at 4°C. The supernatant was taken for further analysis. The content of cecal SCFAs was determined by gas chromatography (GC) using Agilent HP-INNOWAX column (30 m × 0.25 mm × 0.25 μm) in Wuhan GeneCreate Biological Engineering Co. Ltd. (Wuhan, China). The GC conditions were as follows: injector temperature of 250°C; ion source temperature of 300°C; and transmission temperature of 250°C. The temperature program was as follows: initial temperature was set at 90°C, increasing 10°C/min to 120°C; increasing 5°C/min to 150°C; increasing 25°C/min to 250°C; and 250°C for 2 min. Carrier gas, He; flow rate, 1.0 ml/min.

### 2.6 Statistical analysis

All the results in this experiment were expressed as the means ± SEM. The data processing proceeded using SPSS 26.0 software, and variance analysis and the difference between treatments were performed using one-way ANOVA and Duncan's method, respectively. *P* ≤ 0.05 was considered a significant difference. The GraphPad Prism 9.5 software was used to draw the histogram.

## 3 Results

### 3.1 Effects of quercetin and daidzein on egg quality in layers

Eggshell thickness and egg yolk height in weeks 4 and 10, eggshell strength and albumen height in week 4, and egg yolk diameter in week 10 were significantly increased in the quercetin treatment (*P* ≤ 0.05); eggshell thickness in weeks 4 and 10 and egg yolk height in week 10 were significantly increased in the daidzein treatment (*P* ≤ 0.05), compared with control. Egg yolk height and eggshell thickness in week 4 were significantly increased in the quercetin treatment (*P* ≤ 0.05; Table 2), compared with the daidzein treatment.

**Table 2:** Effects of quercetin and daidzein on egg quality in layers.

**Items**	**Weeks**	**Control**	**Quercetin (500 mg/kg)**	**Daidzein (30 mg/kg)**	**SEM**	***P*-value**
Eggshell strength (*N*)	4	34.11^b^	37.57^a^	36.66^ab^	1.50	0.062
	10	32.30	34.13	32.44	1.91	0.568
Egg weight (g)	4	61.67	61.30	61.31	1.22	0.942
	10	59.71	60.70	62.19	1.34	0.186
Eggshell thickness (mm)	4	0.33^c^	0.39^a^	0.37^b^	0.01	< 0.01
	10	0.38^b^	0.41^a^	0.40^a^	0.01	< 0.01
Egg yolk height (mm)	4	15.69^b^	16.71^a^	15.86^b^	0.03	< 0.01
	10	14.51^b^	15.57^a^	15.46^a^	0.03	0.002
Egg yolk diameter (mm)	4	42.71	43.02	42.83	0.40	0.737
	10	40.81^b^	42.25^a^	41.45^ab^	0.49	0.015
Albumen height (mm)	4	3.51^b^	4.11^a^	3.87^ab^	0.25	0.057
	10	3.62	3.95	3.84	0.28	0.478
Egg yolk color	4	8.77	8.53	8.77	0.25	0.572
	10	8.40	8.47	8.27	0.28	0.772
Haugh unit	4	51.06	56.80	55.33	3.00	0.145
	10	53.29	55.44	54.17	3.20	0.796

^a, b, c^Means in the same row with different letters are significantly different (*P* < 0.05). n = 6.

SEM, standard error of the mean.

### 3.2 Effects of quercetin and daidzein on MDA and lipid content in egg yolk of layers

**Figure 1:**
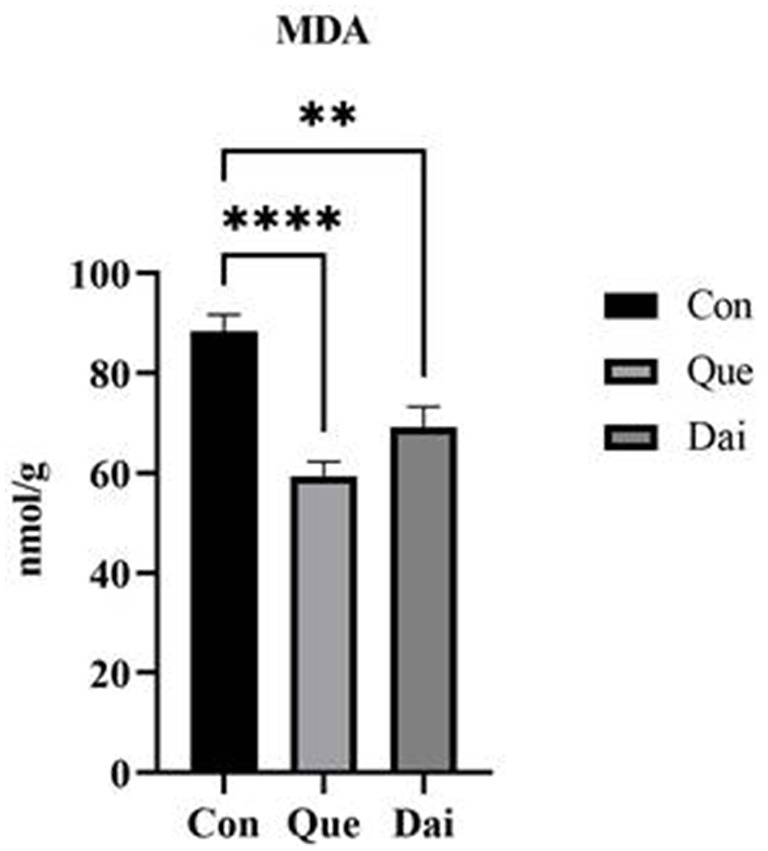
Effects of quercetin and daidzein on MDA content in egg yolk of layers. The results are expressed as mean ± SEM (*n* = 6). ** and **** indicate extremely significant difference (*P* < 0.01). MDA, malondialdehyde.

The contents of MDA, TC, and TG in the egg yolk of the quercetin and daidzein treatments were significantly decreased (*P* ≤ 0.05), the contents of PL and LEC in the egg yolk of the quercetin treatment were significantly increased (*P* ≤ 0.05), compared with control. The contents of TC, TG, and PL, LEC in egg yolk of the quercetin treatment were significantly decreased and increased, respectively, compared with the daidzein treatment (*P* ≤ 0.05; Table 3, Figure 1).

**Table 3:** Effects of quercetin and daidzein on lipid content in egg yolk of layers.

**Items**	**Control**	**Quercetin (500 mg/kg)**	**Daidzein (30 mg/kg)**	**SEM**	***P*-value**
TC (μmol/g)	69.21^a^	43.32^c^	50.48^b^	2.48	< 0.01
TG (μmol/g)	69.66^a^	51.38^c^	59.45^b^	2.43	< 0.01
PL (μg/g)	937.73^b^	1,286.92^a^	1,028.31^b^	57.83	< 0.01
LEC (nmol/g)	326.28^b^	461.52^a^	349.14^b^	22.00	< 0.01

^a, b, c^Means in the same row with different letters are significantly different (*P* < 0.05). n = 6.

SEM, standard error of the mean.

TC, total cholesterol; TG, triglyceride; PL phospholipid; LEC, lecithin.

### 3.3 Effects of quercetin and daidzein on lipid content in the serum and liver of layers

**Figure 2:**
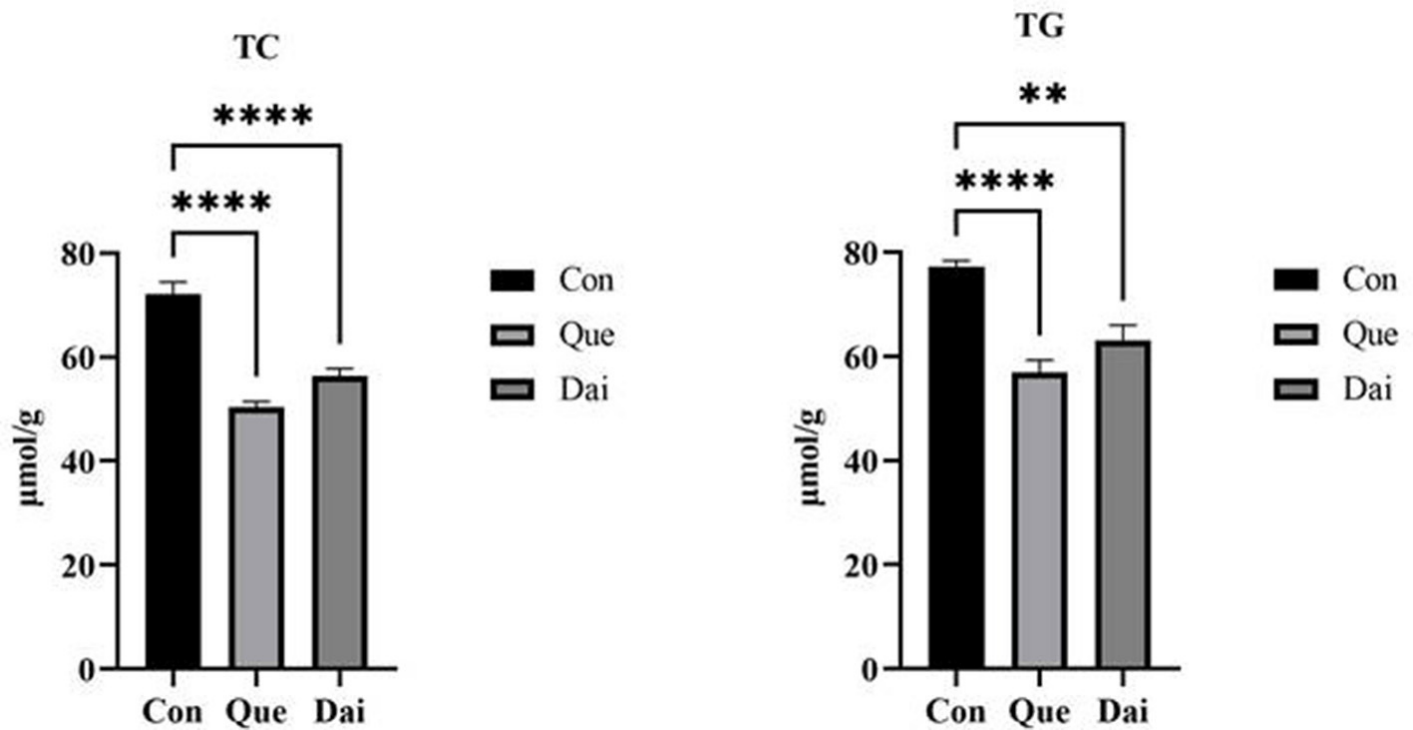
Effects of quercetin and daidzein on lipid content in liver of layers. The results are expressed as mean ± SEM (*n* = 6). ** and **** indicate extremely significant difference (*P* < 0.01). TC, total cholesterol; TG, triglyceride.

The contents of TC and TG in the serum and liver of the quercetin and daidzein treatments were significantly decreased (*P* ≤ 0.05); the VLDL content was significantly decreased and HDL content was significantly increased in the serum of the quercetin and daidzein treatments (*P* ≤ 0.05), and LDL content was significantly decreased in the serum of the quercetin treatment (*P* ≤ 0.05), compared with the control. Contents of TG, VLDL, and LDL were significantly decreased in the serum of the quercetin treatment (*P* ≤ 0.05), and TC content was significantly decreased in the serum and liver of the quercetin treatment, compared with the daidzein treatment (*P* ≤ 0.05; Table 4, Figure 2).

**Table 4:** Effects of quercetin and daidzein on lipid content in serum of layers.

**Items**	**Control**	**Quercetin (500 mg/kg)**	**Daidzein (30 mg/kg)**	**SEM**	***P*-value**
TC (mmol/L)	8.32^a^	6.85^c^	7.60^b^	0.29	< 0.01
TG (mmol/L)	10.06^a^	7.00^c^	8.15^b^	0.36	< 0.01
HDL (μmol/L)	33.09^c^	59.33^a^	52.14^a^	3.46	< 0.01
LDL (mmol/L)	8.37^a^	6.86^b^	7.71^a^	0.34	0.002
VLDL (mmol/L)	8.29^a^	6.79^c^	7.55^b^	0.29	< 0.01

^a, b, c^Means in the same row with different letters are significantly different (*P* < 0.05). n = 6.

SEM, standard error of the mean.

TC, total cholesterol; TG, triglyceride; LDL, low-density lipoprotein; HDL, high-density lipoprotein; VLDL, very-low-density lipoprotein.

### 3.4 Effects of quercetin and daidzein on cecal short-chain fatty acids in layers

The contents of isobutyric acid and valeric acid in the cecum of the quercetin treatment were significantly increased (*P* ≤ 0.05); however, the content of short-chain fatty acids was not affected by daidzein (*P* > 0.05), compared with the control. The content of valeric acid was significantly increased in the cecum of the quercetin treatment (*P* ≤ 0.05), compared with the daidzein treatment (Table 5).

**Table 5:** Effects of quercetin and daidzein on cecal short-chain fatty acids in layers.

**Items**	**Control**	**Quercetin (500 mg/kg)**	**Daidzein (30 mg/kg)**	**SEM**	***P*-value**
Acetic acid (μg/g)	595.77	816.75	749.52	121.55	0.210
Propionic acid (μg/g)	395.24	499.18	478.49	100.58	0.562
Isobutyric acid (μg/g)	32.81^b^	51.44^a^	40.20^ab^	5.81	0.019
Butyric acid (μg/g)	127.38	176.32	132.69	38.64	0.403
Isovaleric acid (μg/g)	29.05	43.21	31.32	6.42	0.092
Valeric acid (μg/g)	38.47^b^	68.61^a^	45.10^b^	8.38	0.007
Caproic acid (μg/g)	1.22	1.49	1.41	0.16	0.234

^a, b^Means in the same row with different letters are significantly different (*P* < 0.05). n = 6.

SEM, standard error of the mean.

## 4 Discussion

### 4.1 Effects of quercetin and daidzein on egg quality in layers

Egg quality including eggshell quality and nutrient content in eggs decreased with age in laying hens after the peak laying period (22). Egg quality includes intrinsic quality (egg yolk height, egg yolk diameter, albumen height, egg yolk color, Haugh unit, etc.) and extrinsic quality (eggshell strength, egg weight, eggshell thickness, etc.), which affects the commercial and edible value of eggs. Eggshell strength and thickness are important indicators of eggshell quality. However, the eggshell quality decreases rapidly with age, which results in huge economic losses (23). Albumen height and Haugh unit are the main indicators of albumen quality, the higher the albumen height and the larger the Haugh unit, the better the albumen quality and the fresher the eggs (24, 25). The egg yolk color is related to the pigment component in feed, and the deep color of the egg yolk means the health of the eggs (26). The egg yolk index refers to the ratio of egg yolk height to egg yolk diameter. The larger the egg yolk diameter, the smaller the egg yolk height, and the more fragile the egg yolk.

In the present study, quercetin increased eggshell strength, eggshell thickness, egg yolk height, and albumen height, and daidzein increased eggshell thickness and egg yolk height. Other studies have also shown that flavonoids may improve the egg quality of birds. A previous study on Lohmann Silver laying hens showed that a diet supplemented with 12 g/kg of Mulberry leaf flavonoids significantly increased eggshell strength and egg yolk color (27). Dietary supplementation with 400 mg/kg of quercetin increased the eggshell thickness, egg yolk height, albumen height, and Haugh unit in Tianfu laying hens during the late laying period (28). Our previous studies showed that dietary supplementation with 0.04% quercetin increased eggshell strength, eggshell thickness, and Haugh unit in Hessian laying hens at 39 weeks old (12). Dietary supplementation with 10 mg/kg of daidzein improved eggshell quality by regulating the shell gland genes (29). The eggshell thickness and eggshell strength tend to increase in laying hens fed by a diet supplemented with daidzein (10, 50, and 100 mg/kg) during the late laying period (30, 31). Dietary supplementation with daidzein also increased egg quality in quail during the late laying period (32). These results further confirmed the positive effect of flavonoids on egg quality in laying hens. Additionally, our results indicated that the effect of quercetin on egg quality was better than daidzein in layers.

### 4.2 Effects of quercetin and daidzein on MDA and lipid content in egg yolk of layers

Lipid peroxidation is a process in which biofilms are attacked by reactive oxygen species, and oxidation of polyunsaturated fatty acids produces harmful lipid peroxides including aldehydes, ketones, and acids (33). Lipid peroxidation reduces the quality and nutritive value of eggs and destroys vitamins, essential amino acids, and other nutrients in egg yolk, resulting in undesirable changes during storage and consumption (34). MDA is one of the end products in the process of lipid peroxidation; therefore, the MDA content may reflect the degree of lipid peroxidation in egg yolk (35). Our study found both quercetin and daidzein significantly decreased MDA content and improved the oxidation stability in egg yolk layers. It indicated that quercetin and daidzein inhibited the production of MDA in egg yolk and reduced the degree of lipid peroxidation, thus increasing albumen height and maintaining the freshness and nutritive value of eggs. It was consistent with other research studies, which reported that flavonoids may prevent the formation of hydroxyl radicals and lipid peroxidation, thus reducing MDA content. A diet supplemented with 1 and 3 g/kg of hesperidin (a bio-flavonoid) significantly reduced the MDA content in egg yolk and improved the antioxidant properties of fresh and stored eggs (36). Dietary supplementation with different doses of quercetin (200, 400, and 800 mg/kg) significantly decreased the MDA content in egg yolk with increasing amounts of quercetin; meanwhile, quercetin also improved the oxidative stability of eggs stored for 28 days at room temperature; and the research findings indicated that quercetin may maintain freshness and extend shelf life of eggs (37, 38). Furthermore, improving the serum and liver antioxidant ability of laying hens in our previous study also supported the reduction of egg yolk MDA content in this study (20).

There are almost all lipids in egg yolk (39). The content of phospholipids accounts for approximately 30% of lipids of egg yolk, and phospholipids are the basic components of biofilms (40), mainly including lecithin, cephalin, and phosphoinositide. Lecithin, known as the “third nutrient,” is a polyunsaturated phosphatidylcholine, which is a structural component of biofilms and may promote brain development and improve memory (41). Egg yolk also contains large amounts of cholesterol. Cholesterol is needed by humans for producing cell membranes and manufacturing the bile acids, vitamin D, and sex hormones; however, excessive intake of cholesterol may induce a series of cardiovascular diseases, including hyperlipidemia, hypertension, and heart disease, which damages health (42). Therefore, it is necessary to reduce cholesterol content in egg yolk. Our study found that both quercetin and daidzein decreased the contents of TC and TG, and quercetin also increased the PL and LEC content in egg yolk. These results indicated that flavonoids regulated lipid metabolism, enhanced lipolysis, and reduced cholesterol deposition in egg yolk, thus inhibiting oxidation of lecithin and improving egg nutritive value. Other researchers have found the same results in flavonoids. A diet supplemented with 0.4%−1.2% of Mulberry leaf flavonoids reduced the contents of TC and TG in egg yolk (27). TC content in egg yolk was decreased by dietary supplementation with quercetin and hesperidin (0.5 g/kg) in laying hens at 28 weeks old (43). Our previous studies also found that 0.04% of quercetin significantly decreased the contents of TC and TG and increased the contents of PL and LEC in the egg yolk of laying hens (11–13). Dietary supplementation with 10, 20, and 40 mg/kg of daidzein decreased TC content in egg yolk with increasing dietary daidzein in Hisex laying hens (44).

### 4.3 Effects of quercetin and daidzein on lipid content in the serum and liver of layers

Poor egg quality resulting from lipid metabolism disorders reduced economic returns in laying hens. The liver synthesizes more than 90% of the cholesterol, and it is the main organ for cholesterol synthesis and transports cholesterol rapidly to blood in laying hens (45). Most of the serum cholesterol is transferred to the egg yolk by lipoproteins. LDL may transport cholesterol to tissues around the liver and cholesterol and triglyceride from the serum to the liver for re-circulation or to form bile acids excreted by the body. Lipids are not synthesized in the ovaries of laying hens. VLDL is the main carrier of cholesterol in the serum, takes charge of transferring cholesterol from the serum to the ovary, and then is absorbed by the oocyte to form egg yolk, thus synthesizing 95% of the cholesterol in egg yolk (46, 47). Therefore, the contents of TG, TC, HDL, LDL, and VLDL in the serum were used to estimate whether lipid metabolism is normal in laying hens. In the present study, both quercetin and daidzein decreased the contents of TC and TG in the serum and liver, decreased the contents of LDL and VLDL in the serum, and increased the HDL content in the serum, which suggested that quercetin and daidzein improved lipid metabolism of layers. Studies have shown that dietary supplementation with flavonoids may regulate lipid deposition in the liver, enhance immunity, and improve egg quality of layers (48). Flavonoids may form insoluble complexes with cholesterol in digesta, thereby inhibiting the intestinal absorption of endogenous and exogenous cholesterol. A diet supplemented with 0.5% fermented *Ginkgo biloba* leaves (flavonoids are the main active components) decreased the contents of TC, TG, and LDL-C and increased the HDL-C content in the serum of 49-week-old laying hens, thus reducing cholesterol content in eggs and improving the egg quality (7). Our previous studies found that 0.04 and 0.06% quercetin decreased the contents of TC, TG, and LDL in the serum, regulated cholesterol metabolism, and reduced abdominal fat percentage in broilers (49). Moreover, the contents of the serum and liver TC and TG tend to decrease with dietary supplementation with 10 mg/kg of daidzein in rats (50). A diet supplemented with 0.05% daidzein decreased the contents of TC, TG, and LDL-C in the serum and improved the meat quality of feeder cattle (51). These results indicated that flavonoids inhibited the activity of 3-hydroxy-3-methylglutaryl coenzyme A (HMG-CoA) reductase, whereby cholesterol content was reduced in the serum and liver, thus decreasing cholesterol deposition in egg yolk (52).

### 4.4 Effects of quercetin and daidzein on cecal short-chain fatty acids in layers

The cecum provides a relatively stable environment and has the largest and most complex microbial community in the intestines of poultry. SCFAs are the end product of microbial fermentation in the cecum, protect intestinal health, and serve as a source of energy for the host (5). A healthy intestinal environment may improve intestinal absorption of Ca; dietary Ca is absorbed by the intestines and deposited on the eggshell gland to form eggshells in laying hens (53). Furthermore, SCFAs regulate the balance among fatty acid synthesis, fatty acid oxidation, and lipolysis in the body (54). Almost all SCFAs are rapidly absorbed by enterocytes and transported to the liver and systemic circulation through the portal vein, affecting lipid metabolism in tissues (55). Butyrate regulated lipid metabolism in the liver and acetate reduced cholesterol levels in the serum of humans (56). Propionic acid may reduce cholesterol synthesis in human Caco-2/TC-7 enterocyte cells by inhibiting HMG-CoA reductase (57). In the present study, quercetin significantly increased the contents of isobutyric acid and valeric acid in the cecum, daidzein did not affect the content of cecal SCFAs. Dietary supplementation with 200 mg/kg of baicalin (a kind of flavonoid) reduced the accumulation of epididymal and perirenal fat in mice, produced SCFAs that are good for the intestines, and improved abnormal lipid metabolism (58). Dietary supplementation with genistein increased the content of intestinal SCFAs in mice, which play a key role in maintaining intestinal epithelial barrier homeostasis (59). A diet supplemented with 0.4 mg/kg of quercetin increased the content of SCFAs in the cecum and improved the intestinal health of laying hens challenged by lipopolysaccharide (LPS) (60). Quercetin increased the content of intestinal SCFAs and protected against antibiotic-induced intestinal dysregulation in mice (61). Moreover, the relative abundance of *Lactobacillus* was positively correlated with SCFA content in the cecum of birds (62), this result is consistent with a previous study in the laboratory that the relative abundance of cecal *Lactobacillus* in the quercetin group was higher than that in the daidzein group of laying hens (20). Therefore, we speculated that quercetin regulated lipid metabolism and protected intestinal health, thus increasing intestinal absorption of Ca and improving eggshell quality in layers, and that daidzein was not as effective as quercetin in improving egg quality, which possibly resulted from unaltered SCFA content in the cecum.

## 5 Conclusion

In conclusion, quercetin and daidzein improved egg quality by decreasing MDA content and cholesterol deposition and regulating lipid metabolism in egg yolk of layers. Quercetin worked better than daidzein in improving egg quality under this experimental condition.

## Data availability statement

The original contributions presented in the study are included in the article/supplementary material, further inquiries can be directed to the corresponding author.

## Ethics statement

All procedures used in this study were approved by the Animal Care and Use Committee of the Northeast Agricultural University (NEAUEC20200203). Housing, management and care of the birds confirmed to the guidelines of Agricultural Animal in Agricultural Research and Teaching of Heilongjiang Province (HEI Animal Management Certificate No. 11928). The study was conducted in accordance with the local legislation and institutional requirements.

## Author contributions

JiL: Writing—original draft, Data curation, Formal analysis, Software. JuL: Data curation, Writing—review & editing. SZ: Data curation, Writing—review & editing. YF: Writing—review & editing, Data curation. QY: Writing—review & editing, Data curation. YL: Writing—review & editing, Funding acquisition.
